# ROOT HAIR DEFECTIVE3 Is a Receptor for Selective Autophagy of the Endoplasmic Reticulum in *Arabidopsis*

**DOI:** 10.3389/fpls.2022.817251

**Published:** 2022-02-24

**Authors:** Jiaqi Sun, Weina Wang, Huanquan Zheng

**Affiliations:** Department of Biology, McGill University, Montreal, QC, Canada

**Keywords:** ER, ER stress, ER-phagy, RHD3, Atg8, autophagosomes

## Abstract

ROOT HAIR DEFECTIVE3 (RHD3) is a plant member of atlastin GTPases, which belong to an evolutionally conserved family of proteins that mediate the homotypic fusion of the endoplasmic reticulum (ER). An atlastin in mammalian cells has recently been shown to act as an ER-phagy receptor for selective autophagy of the ER (ER-phagy) during nutrient starvation. Although RHD3 has been indicated to play a role in ER stress response, it is not very clear how RHD3 is involved in the process. In this study, we showed that the *rhd3* mutant is hyposensitive to ER as well as salt stress. We employed an YFP-tagged ER membrane marker YFP-TMC to monitor the efficiency of ER-phagy microscopically and biochemically. We found that *rhd3* is defective in ER-phagy under ER stress. Furthermore, there is an increased association of YFP-RHD3 with ATG8e-marked autophagosomes. YFP-RHD3 is also visible with ATG8e in the vacuole, and there is a breakdown of YFP-RHD3 under ER stress. RHD3 has two putative ATG8 interaction motifs (AIM1-2). We revealed that RHD3 but not RHD3(ΔAIM1) physically interacts with ATG8, a core autophagosomal component that interacts with various receptor proteins to recruit cargos for degradation by selective autophagy. Furthermore, their interaction is enhanced under ER stress. We thus propose that RHD3 acts as an ER-phagy receptor under ER stress to promote ER-phagy in *Arabidopsis*.

## Introduction

The endoplasmic reticulum (ER) is the largest cellular organelle consisting of interconnected tubules and flattened cisternae that stretch throughout the cytoplasm. Highly conserved across the fungal, plant, and animal kingdoms, the ER is the primary site of protein synthesis, translocation, and modification in all eukaryotic cells. Many other cellular organelles are also physically and functionally linked with the ER. During cell development, the ER remodels its shape continuously to achieve its optical quantity and quality (Brandizzi, 2021). In addition, plants are sessile organisms that live under prevailing environmental conditions. At the cellular level, many environmental stresses can disrupt protein folding in the ER leading to ER stress that is potentially lethal to plant growth (Howell, 2013; Afrin et al., 2020). To alleviate ER stress, plant cells use unfolded protein response (UPR), ER-associated degradation (ERAD), and selective autophagy of the ER (ER-phagy). When the ER is under stress, UPR increases and expanses the size of the ER and the capacity of protein folding in the ER (Bernales et al., 2006), meanwhile, ERAD and ER-phagy become very active (Sun et al., 2021). ERAD recognizes and translocates misfolded proteins into the cytosol for degradation (Sun et al., 2021), while ER-phagy selectively transports *via* autophagosomes and degrades unnecessary or dysfunctional ER domains in the lysosome/vacuole to maintain ER homeostasis (Bernales et al., 2006).

Endoplasmic reticulum-phagy is selective and relies on the binding of specific ER-phagy receptors and autophagy modifier proteins such as ATG8 proteins (Stolz et al., 2014), a family of ubiquitin-like proteins required for the formation of autophagosomes. In recent years, several receptors that mediate ER-phagy have been identified in mammalian cells. For example, Sec62, a constituent of the protein translocon acts in the ER-phagy during stress recovery, and CCPG1, an ER-resident Cell-Cycle Progression Gene 1 protein, can serve as an ER-phagy receptor under ER stress (Fumagalli et al., 2016; Smith et al., 2018). FAM134B, a reticulon-like protein, is also described as an ER-phagy receptor specific for ER sheet degradation (Khaminets et al., 2015; Bhaskara et al., 2019; Jiang et al., 2020). Most recently, RTN3, a reticulon that shapes tubular domains of the ER, and ATL3, an atlastin that mediates ER membrane fusion, have been identified as ER-phagy receptors that target ER tubules for degradation (Grumati et al., 2017; Chen et al., 2019) during nutrient starvation.

In plants, a few ER-phagy receptors have also been currently reported. ATG8 interaction protein 1 (ATI1) and 2 (ATI2) can serve as the starvation-specific ER-phagy receptors in *Arabidopsis* (Honig et al., 2012; Wu et al., 2021). AtSec62 (Hu et al., 2020), C53 (a cytosolic ATG8 interactor in *Arabidopsis*) (Stephani et al., 2020), and RTN1 and RTN2 (two reticulons in maize) (Zhang et al., 2020) have also been reported to modulate ER-phagy in plant cells under ER stress. ROOT HAIR DEFECTIVE3 (RHD3) is a plant member of atlastins (Sun and Zheng, 2018). Similar to its mammalian counterparts, RHD3 mediates the homotypic fusion of ER membranes (Hu et al., 2009; Zhang et al., 2013; Qi et al., 2016). Although it has been suggested that RHD3 plays a role in plant response to ER stress (Lai et al., 2014), it is not clear how RHD3 is involved in ER stress response. In this report, we showed that, by using a combined approach of ER-phagy marker-based imaging, biochemistry, and protein-protein interaction, RHD3 acts as an ER-phagy receptor under ER stress to promote ER-phagy in *Arabidopsis*.

## Materials and Methods

### Plant Growth Conditions, Drug, and Salt Treatment

All mutant seeds, including characterized *rhd3-8* (SALK_025215) (Sun et al., 2020b) and *atg5-1* (SAIL_129_B07) (Le Bars et al., 2014), were obtained from the Arabidopsis Biological Resource Center and then genotyped and propagated. Col.0 (*35Spro:YFP-TMC*) and *rhd3-8* (*RHD3pro:YFP-RHD3*) transgenic line was generated as previously described (Sun and Zheng, 2018; Sun et al., 2020b). *rhd3-8* (*35Spro:YFP-TMC*) and *atg5-1* (*35Spro:YFP-TMC*) plants were created by crossing *rhd3-8* and *atg5-1* mutants with Col.0 (*35Spro:YFP-TMC*), respectively. For ER stress analysis, Col.0 and *rhd3-8* seeds were directly sown on the 1/2 MS medium with or without 2 mM dithiothreitol (DTT). For salt treatment, Col.0 and *rhd3-8* seeds were directly sown on the 1/2 MS medium, and the 6-day-old seedlings of Col.0 and *rhd3-8* were then moved and grown for 5 days on the 1/2 MS medium with or without 150 mM NaCl. The survival rate was quantified based on the color of seedlings. Yellow-colored seedlings were deemed dead. For ER-phagy induction, the 7-day-old seedlings of Col.0, *rhd3-8*, and *atg5-1* were moved to the 1/2 MS liquid medium with 10 mM DTT or with the same concentration of DMSO as control for different hours. For the confocal imaging of YFP-TMC and YFP-RHD3 in the vacuole, 0.1 μM ConcA was added.

### Molecular Cloning

The cloning of the entry vector pCR8-RHD3 was described previously (Sun and Zheng, 2018). pCR8-RHD3(ΔAIM1) was cloned through overlap PCR. The coding sequence of ATG8e was cloned into the pGEM-Gate entry vector. The YFP fragment of pEarleyGate 104 was replaced by mNeonGreen or mCherry to generate pEarleyGate 104-mNeonGreen and pEarleyGate 104-mCherry, respectively, with the AQUA cloning assay (Beyer et al., 2015). Through gateway reaction, pCR8-RHD3 and pGEM-ATG8e were cloned into pEarleyGate 104-mNeonGreen and pEarleyGate 104-mCherry to generate mNeonGreen-RHD3 and mCherry-ATG8e, respectively. For split ubiquitin system (SUS)-based yeast two-hybrid (Y2H) constructs, pCR8-RHD3(ΔAIM1) and pGEM-ATG8e were cloned into pNCW-GWRFC and pNX32-DEST to generate Cub-RHD3(ΔAIM1) and NubG-ATG8e, respectively.

### Confocal Microscopy

Microscopy images were acquired with a Leica SP8 point-scanning confocal system equipped with a × 63 oil objective and using 488- (mNeonGreen/YFP) and 552-nm (mCherry) lasers. Two channels were excited sequentially. The emission filter of mNeonGreen/YFP was 490–560 nm, and the emission filter of mCherry was 580–660 nm.

### Protein Interaction Assays

For bimolecular fluorescence complementation (BiFC) assay, RHD3, RHD3(ΔAIM1), ATG8e, and p24 were cloned into the 3-in-1 BiFC vector (Sun et al., 2020b) with the AQUA cloning method (Beyer et al., 2015). Then, agrobacteria containing different constructs were infiltrated into *Nicotiana benthamiana* leaves with OD_600_ = 0.01. Notably, 72 h later, images were captured by the confocal microscope.

The SUS-based Y2H was performed as previously described (Sun et al., 2020a). The AP4 cells expressing Cub-tagged proteins as indicated and the AP5 cells expressing NubG-tagged proteins as indicated were inoculated in Synthetic Complete (SC) selection liquid medium (-Leu for AP4 and -Trp for AP5) overnight at 30°C. Then, AP4 and AP5 cells were mixed and plated on YPD plates for mating. After 6 h, mated cells were streaked on the -LT selection medium and incubated at 28°C for 2 days. The diploid cells (mated cells) were collected, inoculated in the -LT liquid medium, and incubated overnight at 30°C. Then, the cells were collected and resuspended in water. The optical density (OD) values of all the suspensions were adjusted to 1, 0.1, and 0.01 with water. Then, 10 μl per spot was dropped on -LT or -LTH plates for the interaction test. The plates were incubated at 30°C for 3 days.

Co-immunoprecipitation (Co-IP) was performed as previously described (Sun et al., 2020b). The infiltrated *N. benthamiana* leaves were grinded with the extraction buffer [50 mM Tris–HCl pH 7.5, 150 mM NaCl, 10% (v/v) glycerol, and 0.5% IGEPAL^®^ CA-630 (#I8896, Sigma), 1/100 volume of Protease Inhibitor Cocktail (#P9599, Sigma)]. The extract was centrifuged at 17,000 × *g* for 10 min at 4°C, and the supernatant was collected and incubated with green fluorescent protein (GFP)-Trap beads (Chromotek) for 1 h at 4°C. Then, the beads were washed three times, resuspended in 50 μl 2 × SDS-loading buffer, and boiled for 10 min. The solution was centrifuged, and the supernatant was used for Western blot.

### Western Blot

The total protein was extracted with 1 × SDS loading buffer and boiled for 5 min. The samples were loaded on 10% SDS-PAGE gel and transferred to a polyvinylidene difluoride (PVDF) membrane. Western blot was carried out with anti-GFP antibody (Sigma-Aldrich, G1544) at 1:5,000, anti-mCherry (Sigma-Aldrich, AB356482) at 1:5,000, and anti-tubulin (T6074; Sigma-Aldrich; 1:5,000). The secondary goat anti-rabbit IgG-peroxidase (Sigma–Aldrich, A4914-1ML) was used at 1:5,000 dilution, and goat anti-mouse (A4914-1ML; Sigma–Aldrich) was used at 1:10,000 dilution.

## Results

### *rhd3* Mutant Is Defective in Endoplasmic Reticulum-Phagy Under Endoplasmic Reticulum Stress

**FIGURE 1:**
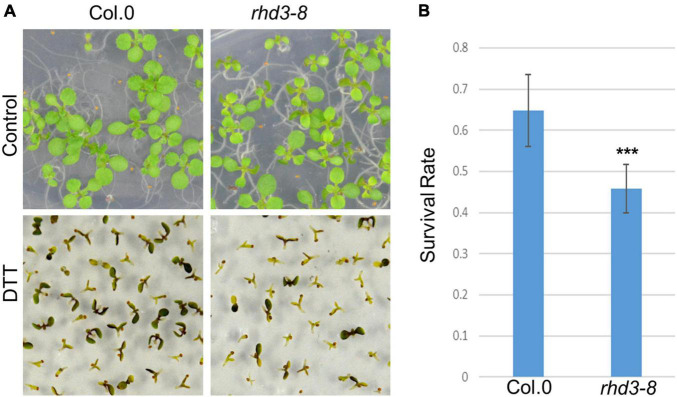
*rhd3* mutant is sensitive to endoplasmic reticulum (ER) stress. **(A)** Ten-day old seedlings of wild type (WT) and *rhd3-8* grown on 1/2 MS in the absence (control) and presence of 2 mM dithiothreitol (DTT). **(B)** Quantification of survival rate of seedlings of WT (*n* = 6, each time with 100–200 seedlings) and *rhd3-8* (*n* = 6, each time with 100–200 seedlings) grown under 2 mM DTT. Error bars represent SD, *** represents significant difference (*P*-value < 0.001, *t*-test).

**FIGURE 2:**
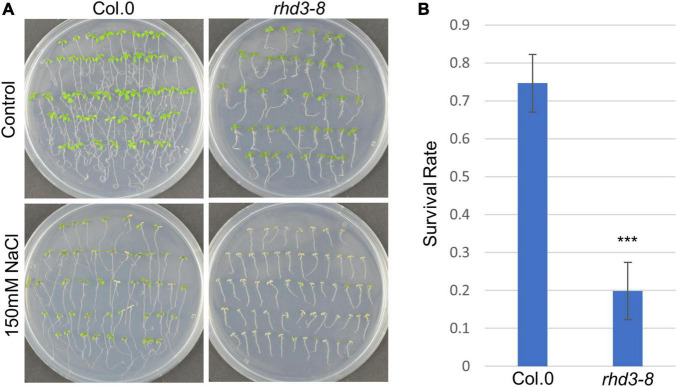
*rhd3* mutant is sensitive to salt stress. **(A)** Six-day old seedlings of WT and *rhd3-8* grown on 1/2 MS for 5 days in the absence (control) and presence of 150 mM NaCl. **(B)** Quantification of survival rate of seedlings of WT (*n* = 4, each time with 40–60 seedlings) and *rhd3-8* (*n* = 4, each time with 40–60 seedlings) grown on 1/2 MS with 150 mM NaCl. Error bars represent SD, *** represents significant difference (*P*-value < 0.001, *t*-test).

To understand how RHD3 may be involved in plant response to ER stress, we first treated seedlings of wild type (WT) and *rhd3-8* mutant with DTT, a reducing agent that blocks disulfide-bond formation in protein folding, thus leading to ER stress. We found that the seedlings of *rhd3-8* were hyposensitive to the treatment of 2 mM DTT, when compared to WT seedlings (Figure 1A). The quantification of the survival rate in the 10-day-old seedlings indicated that the survival rate of *rhd3-8* seedlings was ∼45%, while that of WT was ∼65% (Figure 1B). In addition, the seedlings of *rhd3-8* were also hyposensitive to the treatment of 150 mM NaCl (Figure 2A). The survival rate of *rhd3-8* was ∼20%, while that of WT was above 75% (Figure 2B). These results suggested that the *rhd3* mutant is defective in resolving ER as well as salt stress.

**FIGURE 3:**
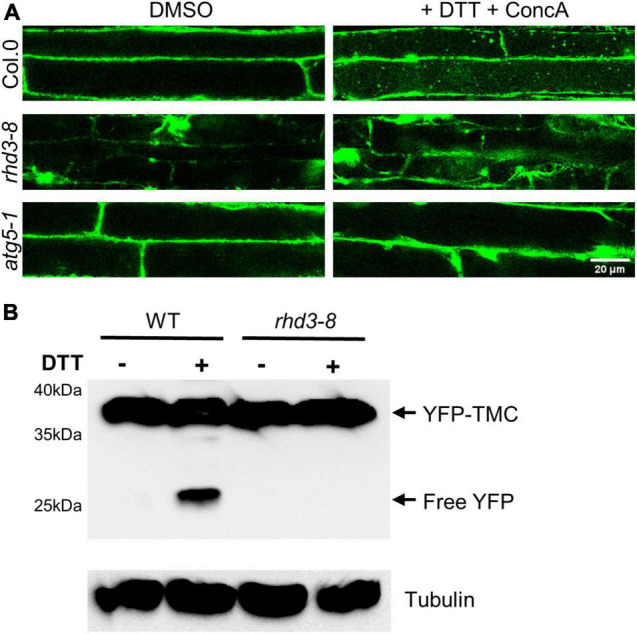
Endoplasmic reticulum (ER)-phagy in *rhd3* mutant is defective. **(A)** Confocal microscopic images of root cells of WT, *rhd3-8*, and *atg5-1* plants expressing YFP-TMC in the absence (DMSO) and presence of 10 mM DTT with ConcA. Scale bars = 20 μm. **(B)** Western blot of YFP-TMC expressed in WT and *rhd3-8* seedlings treated with or without 10 mM DTT for 4 h. Tubulin was used as the loading control.

A strategy that eukaryotic cells use to alleviate ER and other stresses is to activate ER-phagy (Howell, 2013). Therefore, we wondered if the *rhd3-8* mutant is defective in ER-phagy. To test this, we utilized YFP-TMC as a marker to monitor the efficiency of ER-phagy. TMC is composed of the transmembrane (TM) domain and C-terminus of Sey1p (682–766), a yeast homolog of atlastins. When expressed in plant cells, YFP-TMC labels ER membranes without altering the ER morphology and plant growth (Stefano and Brandizzi, 2014; Sun et al., 2020b). It is known that it is difficult to use endogenous ER proteins to monitor ER-phagy as many endogenous ER markers do not change much during ER-phagy, and some ER proteins, e.g., BIP2 and Calreticulin2, are in fact transcriptionally upregulated under ER stress (Srivastava et al., 2018) even they are being degraded by ER-phagy. Exogenously expressed YFP-fused ER proteins do not change their expression significantly, and the cleavage of free fluorescent protein has been utilized as a good biochemical indicator of ER-phagy (Liang et al., 2018). We first monitored the transport of YFP-TMC to the vacuole in the presence of 2 mM DTT with ConcA, a V-ATPase inhibitor that raises the vacuolar pH to stabilize YFP-labeled autophagosomes transported to the vacuole (Liu et al., 2012). As indicated in Figure 3A, in the absence of DTT, there was no much difference between WT, *rhd3-8*, and *atg5-1* mutant cells when the central part of cells was scanned (Figure 3A, the left panel). ATG5 is known to be essential for autophagosome formation and maturation (Le Bars et al., 2014). In 4 h of the presence of DTT, a large number of YFP-TMC bodies were observed in the vacuole of WT plant cells, while no and a few were visible in *atg5-1* and *rhd3-8* mutant cells (Figure 3A, the right panel), respectively. We then examined the cleavage of YFP in YFP-TMC in *rhd3-8* in the absence and presence of DTT by Western blot. As expected, free YFP was detected in WT plants treated with DTT, but no such cleavage was yet detectable in *rhd3-8* plants treated with DTT for 4 h (Figure 3B), although at this time point, there were some visible YFP-TMC punctae in *rhd3-8* under the same conditions (Figure 3A). This indicated that the cleavage of free YFP was compromised in *rhd3-8* plants. Taken all together, we concluded that the *rhd3* mutant is defective in ER-phagy under ER stress.

### ROOT HAIR DEFECTIVE3 Is Increasingly Associated With Autophagosomes Under Endoplasmic Reticulum Stress

**FIGURE 4:**
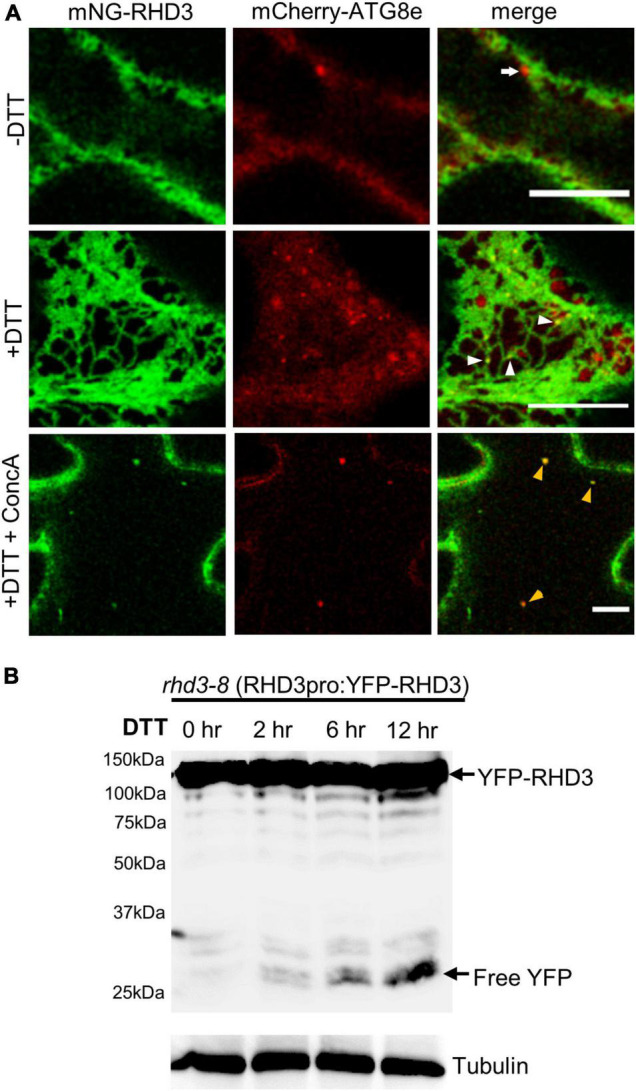
ROOT HAIR DEFECTIVE3 (RHD3) is increasingly associated with autophagosomes under ER stress. **(A)** The association between RHD3 and mCherry-ATG8e-labeled autophagosomes without or with 10 mM DTT treatment. The arrow in the upper panel points to an autophagosome adjacent to mNeonGreen-RHD3 in a cell without the DTT treatment, while the arrowheads in the middle panel point to the increased association of mNeonGreen-RHD3 with autophagosomes in a cell treated with DTT. Yellow arrowheads in the lower panel point to mNeonGreen-RHD3 together with mCherry-ATG8e-labeled autophagosomes in the vacuole. Scale bars = 10 μm. **(B)** Western blot of YFP-RHD3 in *rhd3-8* plants expressing YFP-RHD3 driven by the RHD3 native promoter treated with 10 mM DTT for different hours indicated.

If RHD3 is involved in ER-phagy, there should be an increased association of RHD3 with autophagosomes under ER stress. To test this notion, we transiently co-expressed mNeonGreen-RHD3 and mCherry-ATG8e in tobacco epidermal leaf cells. ATG8e has been widely used as an autophagosomal marker in plants (Zhuang et al., 2017). In cells without DTT, mCherry-ATG8e was largely cytosolic, but mCherry-ATG8e-positive dot-like structures were occasionally observed in close proximity to mNeonGreen-RHD3, which marked ER membranes (Figure 4A, arrow in the upper panel). In cells treated with DTT, however, more mCherry-ATG8e-positive dot-like structures were observed. Many of them were colocalized with mNeonGreen-RHD3 (Figure 4A, white arrowheads in the middle panel). This suggested that RHD3 travels to autophagosomes under ER stress. Furthermore, in the central section of cells treated with DTT and ConcA, mNeonGreen-RHD3 was visible together with mCherry-ATG8e (Figure 4A, yellow arrowheads in the lower panel), indicating that the final destination of mNeonGreen-RHD3 + mCherry-ATG8e is likely the vacuole. We then wondered if there is an increased fluorescent protein breakdown when RHD3 is fused with a fluorescent protein under ER stress. When YFP-RHD3 driven by the RHD3 native promoter (RHD3pro:YFP-RHD3) was expressed in *rhd3-8*, we did not observe any breakdown of YFP-RHD3 in the absence of DTT (Figure 4B, lane 1). However, when *rhd3-8*:RHD3pro:YFP-RHD3 plants were treated with 10 mM DTT, we started to observe a breakdown in 2 h (Figure 4B, lane 2) and a clear breakdown of YFP-RHD3 in 6 h (Figure 4B, lane 3). Taken all together, RHD3 travels together with autophagosomes to the vacuole for degradation under ER stress.

### ROOT HAIR DEFECTIVE3 But Not RHD3(ΔAIM1) Physically Interacts With ATG8, and Such Interaction Is Enhanced Under Endoplasmic Reticulum Stress

**FIGURE 5:**
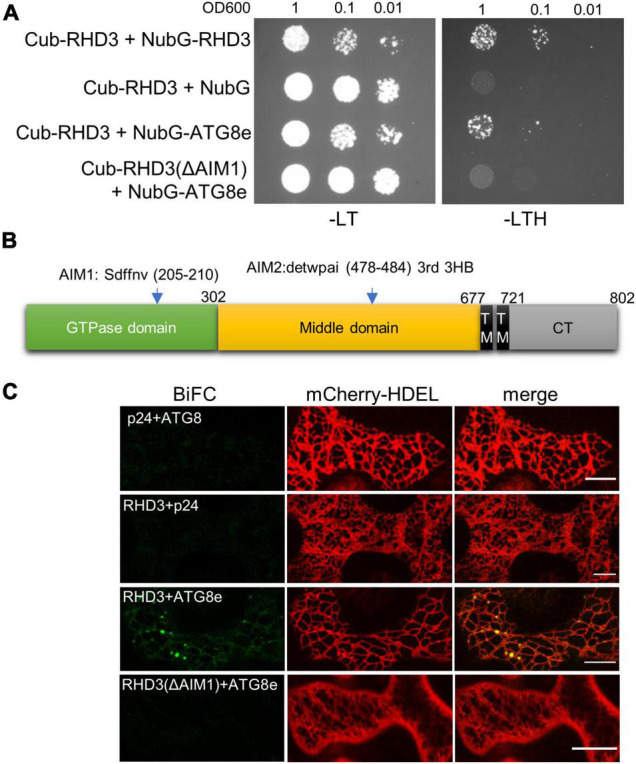
ROOT HAIR DEFECTIVE3 (RHD3) but not RHD3(ΔAIM1) interacts with ATG8. **(A)** The interaction between RHD3/RHD3(ΔAIM1) and ATG8e revealed by the split ubiquitin system (SUS) of yeast two-hybrid (Y2H). RHD3 (row 1) but not RHD3(ΔAIM1) (row 4) interacted with ATG8e. **(B)** The schematic presentation of the locations of two putative AIMs in RHD3 predicted by hfAIM. **(C)** The interaction of RHD3 RHD3(ΔAIM1) and ATG8e in the bimolecular fluorescence complementation (BiFC) system. RHD3 (panel 3) but not RHD3(ΔAIM1) (panel 4) interacted with ATG8e on the ER indicated by mCherry-HDEL. Scale bars = 10 μm.

**FIGURE 6:**
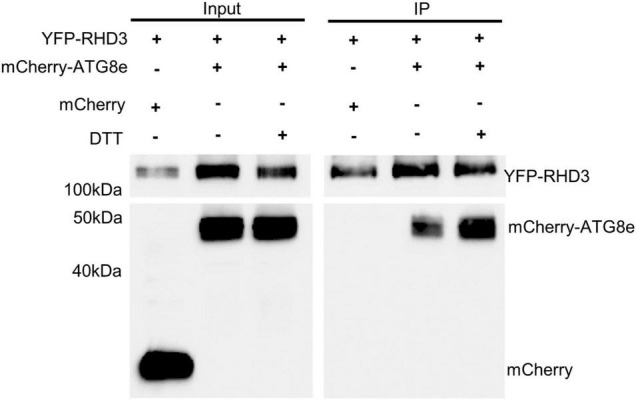
Co-immunoprecipitation (Co-IP) assay of the interaction between RHD3 and ATG8e. mCherrry-ATG8e (lane 2), but not mCherry (lane 1), was copurified by YFP-RHD3. Notably, the increased amount of mCherry-ATG83e was copurified by YFP-RHD3 in the presence of 10 mM DTT (in comparison of lane 3 with lane 2).

ATL3 in mammalian cells has been recognized as an ER-phagy receptor by its interaction with ATG8 (Chen et al., 2019; Molinari, 2021). We thus wondered if RHD3 also acts as an ER-phagy receptor in plants. To investigate this, we examined if RHD3 interacts with ATG8e. In the SUS-based Y2H assay, we found that Cub-RHD3 interacted with NubG-RHD3 (Sun and Zheng, 2018; Figure 5A, the first row, positive control) and NubG-ATG8e (Figure 5A, the third row) but not with NubG (Figure 5A, the second row, negative control). Based on hfAIM, which is a software for the identification of autophagy-associated Atg8-interacting motifs (AIMs) (Xie et al., 2016), RHD3 has two putative AIMs: AIM1 is within the GTPase domain and AIM2 is within the third 3HB in the middle domain (Figure 5B). Because the third 3HB is critical for the stability of RHD3 (Sun and Zheng, 2018), an AIM2 deletion in the 3HB may influence the stability of the protein. We, therefore, tested if RHD3 without AIM1 [RHD3(ΔAIM1)] interacts with ATG8e or not. As indicated, Cub-RHD3(ΔAIM1) did not interact with NubG-ATG8e (Figure 5A, the fourth row). Moreover, in our 3-in-1-based BiFC analysis (Sun et al., 2020a), RHD3, but not RHD3(ΔAIM1), was found to interact with ATG8e on the ER membrane marked by mCherry-HDEL (Figure 5C, the first and fourth panels, respectively). Neither RHD3 nor ATG8e interacted with p24, an ER membrane protein (Chen et al., 2012; Figure 5C, negative controls, the second, and third panels). To further confirm that RHD3 interacts with ATG8 by Co-IP, we transiently co-expressed YFP-RHD3 with mCherry-ATG8e or mCherry in *N. benthamiana* leaves. When YFP-RHD3 was purified by using GFP-Trap beads, mCherry-ATG8e but not mCherry was copurified (Figure 6, compare lane 2 with lane 1). Interestingly, an increased amount of mCherry-ATG8e was copurified when cells were treated with DTT (Figure 6, compare lane 3 with lane 2), suggesting that the DTT treatment enhances the interaction of RHD3 with ATG8 under ER stress.

## Discussion

In this study, we reported that *Arabidopsis* RHD3 can act as an ER-phagy receptor under ER stress and likely under salt stress to promote selective ER degradation by directly interacting with ATG8 *via* its AIM domain. Similarly, atlastin proteins in mammalian cells have been found to act as ER-phagy receptors to regulate ER turnover during nutrient starvation (Liang et al., 2018; Chen et al., 2019). It is interesting to note that mammalian atlastin knockout cell lines are also hyposensitive to ER stress induced by Tunicamycin, a chemical that inhibits protein glycosylation (Zhao et al., 2016), although it has not been reported if atlastins also act as ER-phagy receptors under ER stress. Given what we reported here, it is highly likely that all atlastin proteins can serve as an ER-phagy receptor for the selective degradation of the ER in multiple stress conditions, such as nutrient starvation, salt stress, and ER stress. In plants, it has been found that ATI1 and ATI2 act as ER-phagy receptors in a carbon starvation-induced ER-phagy, and they are highly cargo-specific in the process (Wu et al., 2021). Thus, it will be interesting to examine (1) if RHD3 can serve as an ER-phagy receptor in carbon starvation and/or other environmental stresses such as drought and heat and (2) if RHD3 has its own specific cargos in ER-phagy, and perhaps even its own specific set of different cargos under different environmental stress conditions.

The interaction with ATG8 is one of the key criteria for a protein to be considered as an ER-phagy receptor (Molinari, 2021). Interestingly, RHD3 interacts with ATG8 in the absence of DTT, although the treatment of DTT can enhance the interaction between the two. We attempt to suggest here that RHD3 may also be an ER-phagy receptor for basal ER-phagy. It is known that ER-phagy occurs constitutively at a low level under basal conditions for the turnover of the ER (Khaminets et al., 2015; Wilkinson, 2019).

The RHD3 is known to mediate the homotypic fusion of ER membranes (Hu et al., 2009; Zhang et al., 2013; Qi et al., 2016). How could it also serve as an ER-phagy receptor? In other words, what could turn the action of RHD3 as an ER fusogen to an ER-phagy receptor? In this regard, it is worth noting that the action of C53 as an ER-phagy receptor under ER stress is promoted by an UFMylation E3 ligase UFL1 (Stephani et al., 2021), although the detailed mechanism is not clear. It has been reported that RHD3 interacts with a novel ubiquitination E3 ligase LUNAPARK (LNP) in *Arabidopsis* (Sun et al., 2020a). RHD3 can be ubiquitinated by LNPs (Sun et al., 2020a). It may be that LNPs will sense ER stress and then ubiquitinate RHD3 to promote its action in ER-phagy. It is worth examining (1) if ER stress could promote the ubiquitination of RHD3 by LNPs and (2) if LNP could regulate/enhance the interaction between RHD3 and ATG8. RHD3 is also known to be phosphorylated, i.e., important for the oligomerization of RHD3 (Ueda et al., 2016). This modification could also be a potential regulation in the action of RHD3 in ER-phagy, as the super-assembly of Atg40 (a yeast reticulon-like protein that can serve as an ER-phagy receptor) could induce local ER remodeling to facilitate the autophagosome formation (Mochida et al., 2020).

Finally, it is reported recently that ATL2 and ATL3 in mammalian cells also regulate autophagosome initiation. ATL2 and ATL3 facilitate the recruitment and stabilization of the ULK1 complex onto the ER and tether isolation membranes (an autophagosomal precursor in mammalian cells) with the ER (Liu et al., 2021). The ULK1 complex is crucial for initiating autophagosome formation. Does RHD3 play a similar role in autophagosome initiation in plant cells as well? This can be tested by investigating the action of ATG1 in the *rhd3* mutant. ATG1 (a homolog of ULK1 in *Arabidopsis*) plays a role in autophagy under nitrogen deprivation and short-term carbon starvation (Huang et al., 2019).

## Data Availability Statement

The raw data supporting the conclusions of this article will be made available by the authors, without undue reservation.

## Author Contributions

JS designed and performed all experiments excepting Y2H, collected the data, and wrote the first draft of the manuscript. WW performed the Y2H experiment. HZ conceived the project, analyzed the data, and revised the manuscript. All authors read and approved the final manuscript.

## Conflict of Interest

The authors declare that the research was conducted in the absence of any commercial or financial relationships that could be construed as a potential conflict of interest.

## Publisher’s Note

All claims expressed in this article are solely those of the authors and do not necessarily represent those of their affiliated organizations, or those of the publisher, the editors and the reviewers. Any product that may be evaluated in this article, or claim that may be made by its manufacturer, is not guaranteed or endorsed by the publisher.
